# Physiological response and microRNA expression profiles in head kidney of genetically improved farmed tilapia (GIFT, *Oreochromis niloticus*) exposed to acute cold stress

**DOI:** 10.1038/s41598-017-18512-6

**Published:** 2018-01-09

**Authors:** Jun Qiang, Yan T. Cui, Fan Y. Tao, Wen J. Bao, Jie He, Xia H. Li, Pao Xu, Lan Y. Sun

**Affiliations:** 1 Key Laboratory of Freshwater Fisheries and Germplasm Resources Utilization, Ministry of Agriculture, Freshwater Fisheries Research Centre, Chinese Academy of Fishery Sciences, 9 Shanshui East Road, Wuxi, Jiangsu 214081 China; 2 Wuxi Fisheries College, Nanjing Agricultural University, 9 Shanshui East Road, Wuxi, Jiangsu 214081 China

## Abstract

Cold stress has a serious impact on the overwintering survival and yield of genetically improved farmed tilapia (GIFT, *Oreochromis niloticus*). Understanding the physiological and molecular regulation mechanisms of low-temperature adaptation is necessary to help breed new tolerant strains. The semi-lethal low temperature of juvenile GIFT at 96 h was determined as 9.4 °C. We constructed and sequenced two small RNA libraries from head kidney tissues, one for the control (CO) group and one for the 9.4 °C-stressed (LTS) group, and identified 1736 and 1481 known microRNAs (miRNAs), and 164 and 152 novel miRNAs in the CO and LTS libraries, respectively. We verify the expression of nine up-regulated miRNAs and eight down-regulation miRNAs by qRT-PCR, and found their expression patterns were consistent with the sequencing results. We found that cold stress may have produced dysregulation of free radical and lipid metabolism, decreased superoxide dismutase activity, reduced respiratory burst and phagocytic activity of macrophages, increased malondialdehyde content, and adversely affected the physiological adaptation of GIFT, eventually leading to death. This study revealed interactions among miRNAs and signal regulated pathways in GIFT under cold stress that may help to understand the pathways involved in cold resistance.

## Introduction

Fish are susceptible to environmental factors such as temperature, salinity, pH, dissolved oxygen, ammonia nitrogen, nitrite, and various viruses. Water temperature (low or high) is one of the most important factors because of its obvious affection on immune and antioxidant activity of fish^1–3^. Temperature stress may result in disorders of body physiological homeostasis, which could consequently inhibit survival and growth^4,5^. Low water temperature can have an inhibitory effect on the immune system, which increases the sensitivity of fish to pathogens. For instance, Ndong *et al*.^6^ and Tort *et al*.^7^ found that cold stress suppressed the activities of serum lysozyme (LYZ) in Mozambique tilapia (*Oreochromis mossambicus*) and gilthead bream (*Sparus aurata*) and, in Mozambique tilapia, the pathogenicity of *Streptococcus iniae* increased^6^. He *et al*.^8^ and Liu *et al*.^9^ found that acute cold stress increased serum cortisol levels and inhibited LYZ activity and decreased complement and thyroxine levels in genetically improved farmed tilapia (GIFT, *Oreochromis niloticus*). Antioxidant defence systems are present in all aerobic organisms, and their main function is to remove excess free radicals and other reactive oxygen species (ROS) to protect cells from damage^10^. In appropriate amounts, free radicals can have a positive effect on organisms because they participate in energy synthesis, phagocytosis of bacteria, regulation of cell growth, and cell signalling^10,11^. However, in fish under cold stress, an excess of free radicals causes oxidative damage by reacting with polyunsaturated fatty acids in biofilms and increasing lipid peroxides (e.g. malondialdehyde, MDA)^2^.

The tolerance of teleost to low temperature may be related to fish size^12^, strain^13^, nutritional status^14^, or stress conditions^8^. For example, Sogard^12^ reported that mortality was associated with the size of both freshwater and marine teleost fish size during overwintering; smaller fish were less cold tolerant than the larger fish in the same strain. When four different strains of tilapia (average size 50.73 g ± 4.23) were subjected to cold stress with water temperature reduced from 26 °C to 8 °C at a rate of 3 °C/d, He *et al*.^13^ found that the cold tolerance of GIFT and red tilapia (*O*. *niloticus* × *O. mossambica*) was significantly lower than that of hybrid tilapia (*O*. *niloticus* × *O*. *aureus*) and blue tilapia (*O*. *aureus*). Increasing the protein levels of feed can also significantly increase the cold tolerance of GIFT^14^.

At present, the winter temperature is low in the main tilapia-rearing areas of China and, in recent years, more cold waves have occurred with temperatures that are often below 10 °C and the low temperature duration has been longer, which greatly affects the survival and production of tilapia. It was found that tilapia generally stopped feeding when the water temperature was below 18 °C and began to die when the water temperature was below 14 °C or 12 °C (depending on the tilapia species and stress time)^15,16^. After the introduction of GIFT by the World Fisheries Centre in 2006, we conducted further breeding programs to select for growth characteristics and resistance to stress. We found that GIFT bred in water at 15 °C fed normally, and death occurred when the water temperature was below 11 °C^13^. GIFT has become the main tilapia species that is bred in southern China and, as such, plays an important role in improving fishery production and increasing fishers’ income. However, few studies have focused on the regulation and response mechanisms of GIFT under cold stress. Understanding the low temperature adaptation mechanism of GIFT can not only reduce the over-winter mortality and improve the efficiency of farming, but also provide theoretical support for breeding new strains with low temperature resistance.

MicroRNAs (miRNAs) are non-coding RNAs, 18–25 nucleotides in length, that regulate the transcription levels of their target genes. They are widely involved in physiological processes such as regulation of osmotic pressure, reproduction, development, growth, immune systems, and metabolism^17–21^. Hung *et al*.^22^ found that in zebrafish (*Danio rerio*) exposed to 18 °C cold stress for 4 h, 29 miRNAs were up-regulated and 26 miRNAs were down-regulated by small RNA sequencing. Gene ontology enrichment analysis showed that these miRNAs were involved mainly in melanogenesis, the GnRH pathway, and circadian rhythm. Yang *et al*.^23^ reported 25 miRNAs that were differentially expressed in brain tissue of zebrafish cold-acclimated at 10 °C for 10 d compared with a control group. However, in brain tissue, miRNA-mediated gene regulation may play only a minor role in the thermal adaptability of fish under cold stress.

In teleost fish, head kidney tissue has a similar role as the mammalian adrenal gland and is involved in physiological responses to stress^24^. Head kidney is extremely sensitive to changes in the external environment and plays a central role in the coordination and regulation of metabolism, immunity, and endocrine systems in response to stress^25,26^. Head kidney tissue is also an important biological target organ that has been used to monitor stress in response to changing environmental conditions^8,20^. However, it is still unclear whether the head kidney tissue of tilapia plays a role in cold adaptability and response regulation. To investigate this problem, we used biochemical methods to determine superoxide dismutase (SOD) and LYZ activity, and MDA, levels in the head kidney of GIFT post-cold stress. Macrophages were isolated from head kidney, and the respiratory burst activity (RBA) and phagocytic activity (PA) of the macrophages were measured. We also constructed small RNA libraries for a low temperature stress group and a control group and sequenced them using high-throughput sequencing technology. We identified miRNAs that were differentially expressed between the two libraries and predicted the biological pathways that they may regulate. The findings of the present study reveal novel insights about the molecular mechanisms that mediate the cold stress-induced response in GIFT head kidney.

## Material and Methods

### Ethics statement

All the methods used in this study were performed in accordance with the Guidelines for Experimental Animals established by the Ministry of Science and Technology (Beijing, China). The study protocols were approved by the Freshwater Fisheries Research Centre at the Chinese Academy of Fishery Sciences (Wuxi, China). Head kidney was extracted based on the Guide for the Care and Use of Laboratory Animals in China.

### Experimental fish

The same batch of breeding fry at 10 days after hatching were obtained from the Yixing Experimental Base of the Freshwater Fisheries Research Centre at the Chinese Academy of Fishery Sciences and cultured in an outdoor cement pond for 30 days. The fish were returned to the laboratory and placed in a water-cycling system. The fish were acclimated at 28 °C for 3 weeks using an automatic temperature controller. About 850 60-day-old juvenile GIFT were used in this study. The fish were fed twice a day (7:00 and 16:00) with a diet containing 30% protein and 8% fat. The average weight of the experimental juveniles was 105.58 ± 5.43 g. The fish were fasted for 24 h before the start of the study.

### Determining the 96-h median lethal low temperature in GIFT

The 96-h median lethal low temperature (96h-LT_50_) of GIFT was determined by setting five low temperature conditions as 12, 11, 10, 9, and 8 °C. Through the circulating water cooling system, the water temperature, which started at 28 °C, was dropped sharply at 8–10 °C/h to reach each of the experimental temperatures after 2 h. Five treatments with three replicates (each with 10 fish, making a total of 150 fish) were set up. The cumulative mortality of each treatment group within 96 h was counted and the 96h-LT_50_ of the GIFT was obtained by linear interpolation. The 96h-LT_50_ was used as the experimental water temperature. During the experiment, dissolved oxygen was higher than 5 mg L^−1^, pH was 7.6 ± 0.2, and ammonia nitrogen and nitrite levels were lower than 0.1 mg L^−1^.

### Treatment and sampling

A total of 660 experimental fish were used for the actual experiment. The experimental methods and management were as described above.

#### Experiment one

Sixty experimental fish were placed randomly in six tanks (10 fish per tank); three 96h-LT_50_ tanks (LTS group) and three 28 °C control tanks (CO group). The cumulative mortality in each tank was calculated at 0, 2, 6, 12, 24, 48, and 96 h.

#### Experiment two

Six hundred experimental fish were placed randomly in 12 tanks (50 fish per tank); six 96h-LT_50_ tanks (LTS group) and six 28 °C control tanks (CO group). Nine samples were collected from three LTS tanks and three CO tanks (three fish from each tank) at each sampling time respectively. The sampling times were 0, 2, 6, 12, 24, 48, and 96 h. Three fish were randomly captured from each tank and rapidly anesthetized with 200 mg L^−1^ MS-222. The head kidney tissues were removed, divided into two parts, frozen with liquid nitrogen, and stored at −80 °C. One part of the sample was used to measure SOD and LYZ activity and MDA content as follows. The head kidney was homogenized as described by He *et al*.^2^. SOD activity was determined as described by Beauchamp *et al*.^27^. MDA content was measured as described by Zhang *et al*.^28^. LYZ activity was measured as reported by Hultmark *et al*.^29^. All the required kits were purchased from the Nanjing Jiancheng Biological Engineering Institute (Nanjing, China), and were used according to the manufacturer’s instructions. The other part of the sample was used to analyse the expression levels of the miRNAs and their genes at the different sampling times by qRT-PCR.

Another nine samples were collected from the other three LTS tanks and other three CO tanks (three fish from each tank) at each of the sampling times and anesthetized as described above. The head kidney tissues were removed under sterile conditions. Isolation, purification, and culture of the macrophages from the head kidney samples were as described by Chen *et al*.^30^. We used KOH/DMSO as a blank control. Absorbance was measured at 630 nm using a BioTek Synergy H1 Multi-Mode Reader (Vermont, USA) to determine the RBA of the head kidney macrophages^31^. The method used to measure the PA of the macrophages was as described by Pulsford *et al*.^32^.

### Construction and sequencing of small RNA libraries from head kidney of GIFT exposed to 96h-LT_50_ stress by high-throughput sequencing

After 24 h of exposure to 96h-LT_50_ stress (LTS group) or to 28 °C (CO group), one fish was randomly captured from each tank; one from each of the six LTS tanks and one from each of the six CO tanks making a total of 12 fish. The head kidney tissues were removed as described above. Total RNA was extracted from the head kidney samples using Trizol reagent (Invitrogen, CA, USA) following the manufacturer’s instructions. The quantity and purity of the extracted RNA were measured using a RNA 6000 Nano Lab Chip Kit and a Bioanalyzer 2100 (Agilent, CA, USA), respectively. The samples with an RNA integrity number >7.0 were used for sequencing. Equal amounts of total RNA from the six CO samples or six LTS samples were mixed and pooled separately to build two small RNA libraries. The CO and LTS libraries were sequenced and the reads were assembled following standard procedures^33^.

The basic sequencing data and identification of conserved and novel miRNAs, as well as differentially expressed miRNAs were analysed according to Qiang *et al*.^34^. Briefly, the raw data were filtered by removing common RNA families (rRNA, tRNA, snRNA, snoRNA), adapter sequences, and sequences with low complexity and repeats. Then, the remaining small RNA sequences of 18–30 nt that matched the Nile tilapia reference genome (http://www.ncbi.nlm.nih.gov/genome/?term=Oreochromis%20niloticus) were compared against all the known mature miRNA sequences of animals in miRbase Release 21.0 (http://www.mirbase.org/) by BLAST searches. Small RNAs with less than two mismatches outside the seed region were considered as conserved miRNA. The remaining small RNAs that did not match known miRNA sequences were mapped to the reference genome. Their flanking sequences were obtained and used to predict the stem-loop structures of the candidate miRNA precursor sequences. Sequences that formed a stable stem-loop structure were considered to be novel 5p- or 3p- derived miRNAs.

### Validation of differentially expressed miRNAs in GIFT head kidney after 24 h of 96h-LT_50_ stress by qRT-PCR

Total RNA was extracted from head kidney tissue samples from GIFT exposed to 96h-LT_50_ cold stress (LTS group) or to 28 °C (CO group) for 24 h; two samples from each of the six CO and six LTS tanks, using Trizol reagent (Invitrogen, CA, USA). A Mir-X™ miRNA First-Strand Synthesis kit and a SYBR^®^ PrimeScript^TM^ miRNA RT-PCR kit (Takara, Dalian, China) were used for the RT reaction and qRT-PCRs of the miRNAs as described previously^34^. The relative fold changes in the expression levels of the miRNAs relative to the expression of U6 (used as the reference gene) were calculated by the 2^−ΔΔCt^ method. The miRNA specific primers were synthesized by Genewiz, Inc. (Genewiz, Suzhou, China).

### Expression profiles of some miRNAs and their target genes in head kidney of GIFT under 96h-LT_50_ stress

Head kidney tissue samples were taken from the GIFT in Experiment two. Total RNA extraction, and the RT reaction and qRT-PCRs of the miRNAs were as described above. We used the miRanda v3.3a toolbox (http://www.microrna.org/microrna/home. do) and TargetScan 5.2 (http://www.targetscan.org/vert_50/) to predict the potential target genes of selected differentially expressed miRNAs using a procedure based on Qiang *et al*.^34^. The RT reaction and qRT-PCRs of the predicted target mRNAs were measured using PrimeScript™ RT Master Mix and SYBR^®^ Premix Ex Taq kits (Takara, Dalian, China), as described in our previous study^21^. 18 S rRNA was used as a reference. The mRNA and 18 S rRNA primers were synthesized by Shanghai GeneCore Bio Technologies Co., Ltd. (Shanghai, China) (Table 1). The measurement of miRNA expression was as described above. The results were used to analyse the expression levels of the miRNAs and their potential target genes in GIFT under 96h-LT_50_ cold stress.

**Table 1:** Primer design of miRNA and mRNA.

Name	Primer sequence (5′-3′)
miR-1	CCGCGTGGAATGTAAAGAAGT
miR-106a	AAGCGACCTAAAGTGCTTACAG
miR-143	AACACGCTGAGATGAAGCACT
miR-122	CTGGAGTGTGACAATGGTGTTT
miR-192	AACACGCTGACCTATGAATTG
miR-462	AAGCGACCGTAACGGAACCCAT
miR-29a	GCACCATTTGAAATCGGTTAG
18 s rRNA	F: 5′-GGCCGTTCTTAGTTGGTGGA-3′
	R: 5′-TTGCTCAATCTCGTGTGGCT-3′
SCD	F: 5′-ACAAGCTCTCCGTGCTGGTCAT-3′
	R: 5′-GCAGAGTTGGGACGAAGTAGGC-3′
SIRT1	F: 5′- TCCAGATATCCCCCTGGCAA-3′
	R: 5′- CAGGAGCTCGACGTCTCATC-3′
TPMT	F: 5′- GTGTTCATTCAGGAAAGGTGCT-3′
	R: 5′- CACTGTCGCTGTAGGTCTGT-3′
PPP1R12B	F: 5′-CCACTACTAAGAGCCTGCGG-3′
	R: 5′- CCTGTCTCCCCTTCTTTTTATAGT-3′
MBNL1	F: 5′-CTGTCACCTTTGTTGCCTGC-3′
	R: 5′- TGGTGTCACCGAGAACATGG-3′

### Data analysis

Data were expressed as mean ± standard error of mean (SEM). The data were analysed by one-way analysis of variance (ANOVA) using the SPSS 17.0 software. *P* values < 0.05 were considered statistically significant.

###  Data availability

All data supporting the conclusions of this article are included within the article.

## Results

### Assessment of 96h-LT_50_ cold stress in GIFT

**Figure 1:**
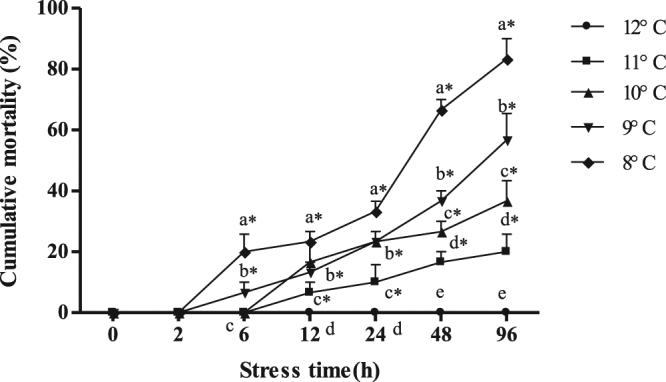
Cumulative mortality in GIFT under cold stress at different sampling points. *Indicates significant differences (*P* < 0.05) between values obtained before and after cold-stress, by paired-samples t-test. Diverse lowercase letters show significant differences (*P* < 0.05) in different groups at each sampling point, by Duncan’s multiple range test.

The stress temperatures used in Experiment one had significant effects on GIFT survival (Fig. 1). At 12 °C, there were no GIFT deaths at 96 h. At 11 °C, GIFT began to die at 12 h and the cumulative mortality was 20% at 96 h. At 9 °C and 8 °C, the cumulative mortalities at 12 h were 6.7% and 20%, respectively; at 24 h, they were 23.3% and 33.3%, respectively; and at 48 h and 96 h, the cumulative mortalities were 56.7% and 83.3%, respectively. Thus, after 96 h of exposure to cold stress, the cumulative mortality of the GIFT exposed to 8 °C stress was significantly higher than the cumulative mortality of the GIFT in the other cold-exposed groups (*P* < 0.05). The 96h-LT_50_ calculated by linear interpolation was 9.4 °C [y = 242.64 − 20.33 × (R = 0.996), *P* < 0.01]. Therefore, we selected 9.4 °C as the 96h-LT_50_ to be used in the subsequent experiments in this study.

### SOD and LYZ activity and MDA content in head kidney of GIFT under 96h-LT_50_ stress

**Figure 2:**
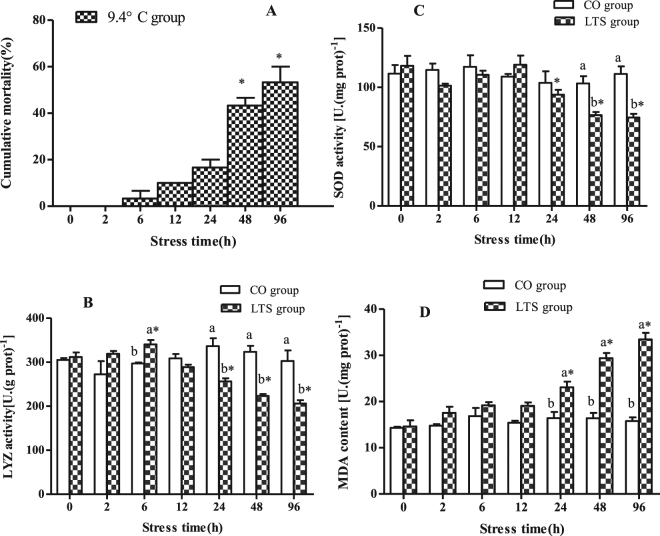
Cumulative mortality, and LYZ and SOD activity, and MDA content in head kidney tissues of GIFT under 96h-LT_50_ stress. (**A**) Cumulative mortality. *Indicates significant differences (*P* < 0.05) among different sampling time points. (**B**) LYZ activity; (**C**) SOD activity; (**D**) MDA content. In (**B**–**D**), *indicates significant differences (*P* < 0.05) between values obtained pre- and post-9.4 °C stress, by paired-samples t-test. Diverse lowercase letters show significant differences (*P* < 0.05) between the control group and 9.4 °C-stressed group at each sampling point, by Duncan’s multiple range test.

Within the 96h-LT_50_ stress period, the GIFT began to show some behavioural and physiological changes. At 6 h, the GIFT began to stop at the bottom of the tanks and swimming was reduced. At 12 h, some fish lay on the tank bottom and when the abdomen of the fish was touched gently, the fish would swim quickly; such fish soon died. At 24 h, GIFT began to die, and at 48 h and 96 h, the mortality rose sharply (Fig. 2A).

LYZ activity in the head kidney of GIFT in the LTS group was significantly higher than in the CO group at 6 h post-9.4 °C stress (*P* < 0.05), but there was no significant difference in LYZ activity between the LTS and CO groups at 12 h post-9.4 °C stress (*P* > 0.05) (Fig. 2B). At 24 h post-9.4 °C stress, the activities of LYZ and SOD decreased significantly (Fig. 2C). At 48 h and 96 h post-9.4 °C stress, the activities of SOD and LYZ were significantly lower in the LTS group compared with the CO group (*P* < 0.05). The MDA contents in the GIFT head kidney was significantly higher in the LTS group compared with the CO group at 24 h post-9.4 °C stress (*P* < 0.05) (Fig. 2D).

### RBA and PA of macrophages in head kidney of GIFT under 96h-LT_50_ stress

**Figure 3:**
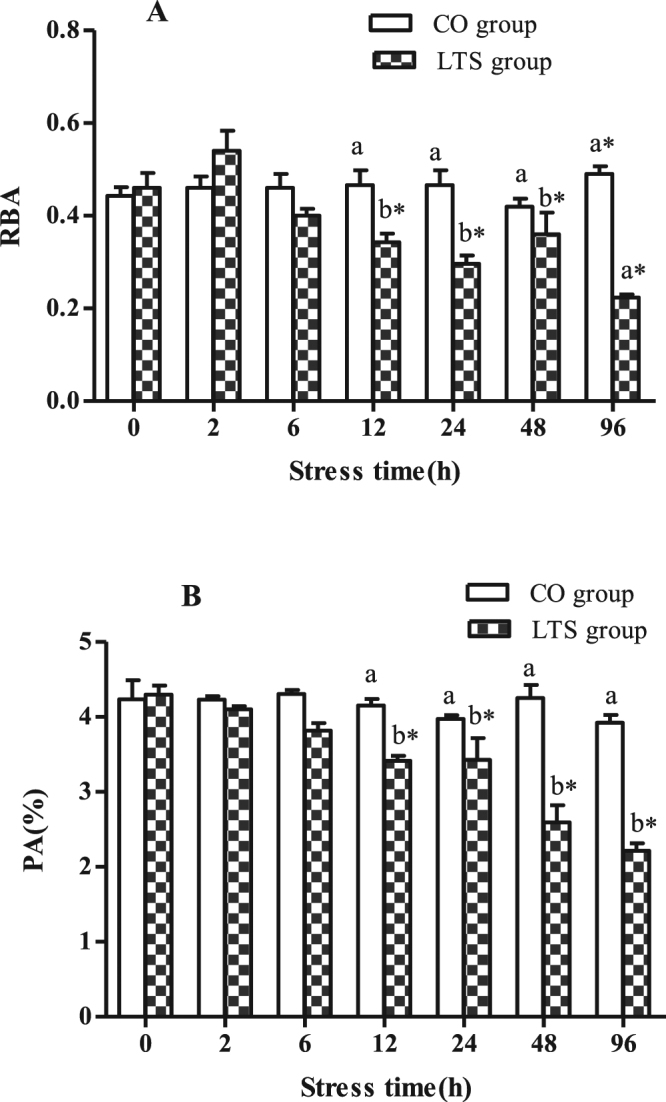
RBA and PA in head kidney macrophages of GIFT under 96 h LT_50_ stress. *Indicates significant differences (*P* < 0.05) between values obtained pre- and post-9.4 °C stress, by paired-samples t-test. Diverse lowercase letters show significant differences (*P* < 0.05) between the control group and 9.4 °C-stressed group at each sampling point, by Duncan’s multiple range test.

At 6 h post-9.4 °C stress, no significant differences were detected in the RBA and PA of the macrophages between the CO and LTS groups (Fig. 3). At 12 h post-9.4 °C stress, the RBA and PA decreased significantly (*P* < 0.05). This observation combined with the changes in SOD and LYZ activities and MDA content, indicated that 24 h under 96h-LT_50_ stress may be an important threshold for the stress response of GIFT. Therefore, we used the head kidney tissues of GIFT at 24 h post-9.4 °C stress to construct the small RNA libraries and analyse the miRNA expression profiles.

### Identification and expression profiling of miRNAs in head kidney of GIFT under 96h-LT_50_ stress

**Figure 4:**
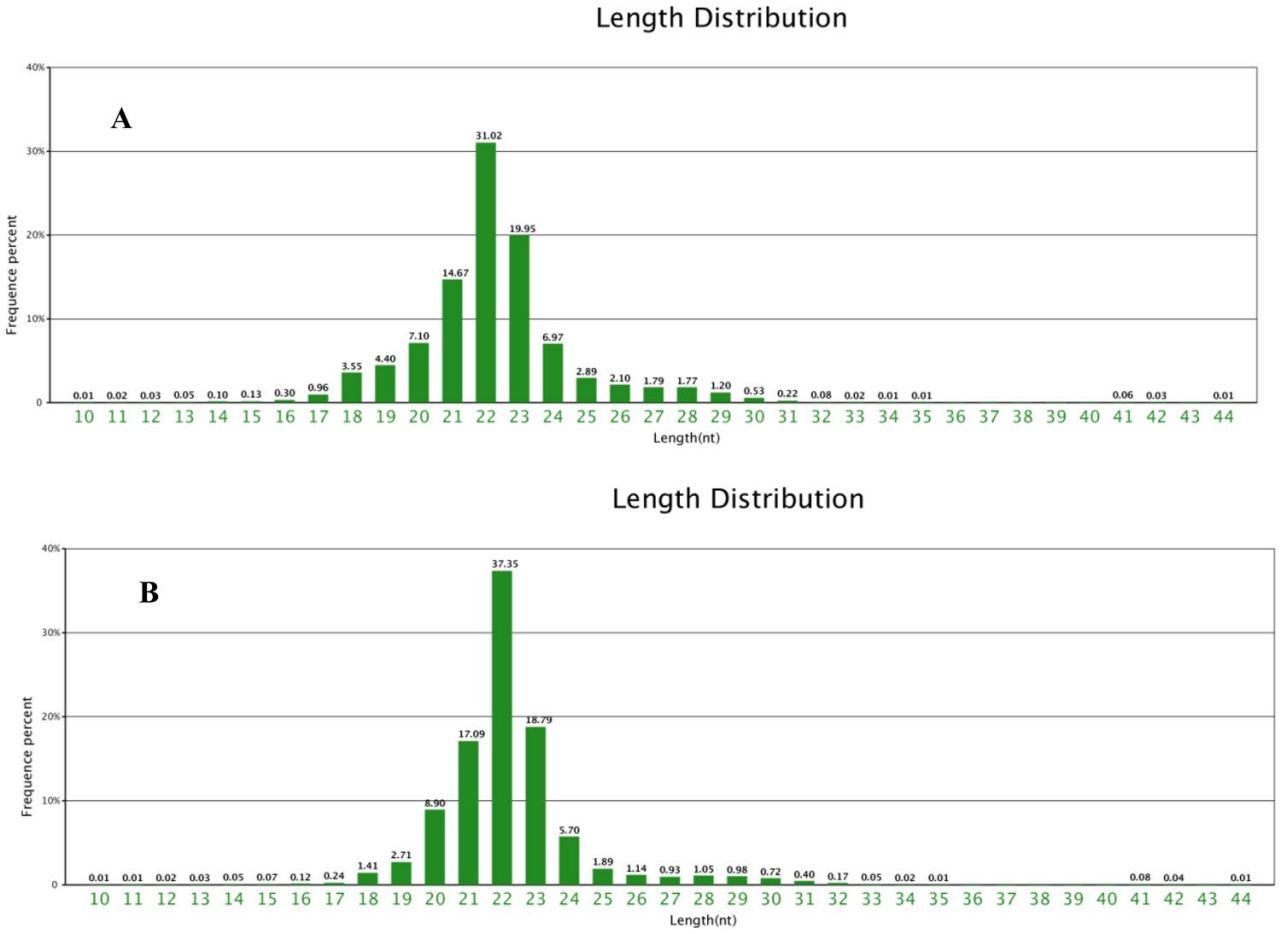
Length distribution of the small RNA sequences in the control group (CO group) (**A**) and 9.4 °C-stressed group (LTS group) (**B**) at 24 h.

Two small RNA libraries (LTS at 9.4 °C and CO at 28 °C for 24 h) were constructed and sequenced by high-throughput sequencing. A total of 492,815 and 541,895 small RNAs (18–30 nt) were obtained in the LTS and CO libraries, respectively (Table 2). Reads of 21–23 nt accounted for 65.64% and 73.23% of the reads in CO and LTS libraries, respectively (Fig. 4). The number of 22-nt long reads was significantly higher than the numbers of 21-nt and 23-nt long reads (*P* < 0.05). The obtained reads were compared with the Nile tilapia reference genome, and 228,075 (46.28%) and 264,661 (48.84%) of the reads were uniquely aligned. We identified 1736 and 1481 known miRNAs in the CO and LTS libraries, respectively. In addition, we predicted 164 and 152 potential novel miRNAs in the CO and LTS libraries, respectively.

**Table 2:** Preliminary analysis of high-throughput sequencing data in two small RNA libraries of GIFT head kidney. CO library, control group at 28 °C; LTS library, 9.4 °C-stressed group.

	CO library	LTS library
Total small RNA(18–30 nt)	541,895	492,815
Mapping to genome	264,661	228,075
Known miRNA	1736	1481
Novel miRNA	164	152

**Figure 5:**
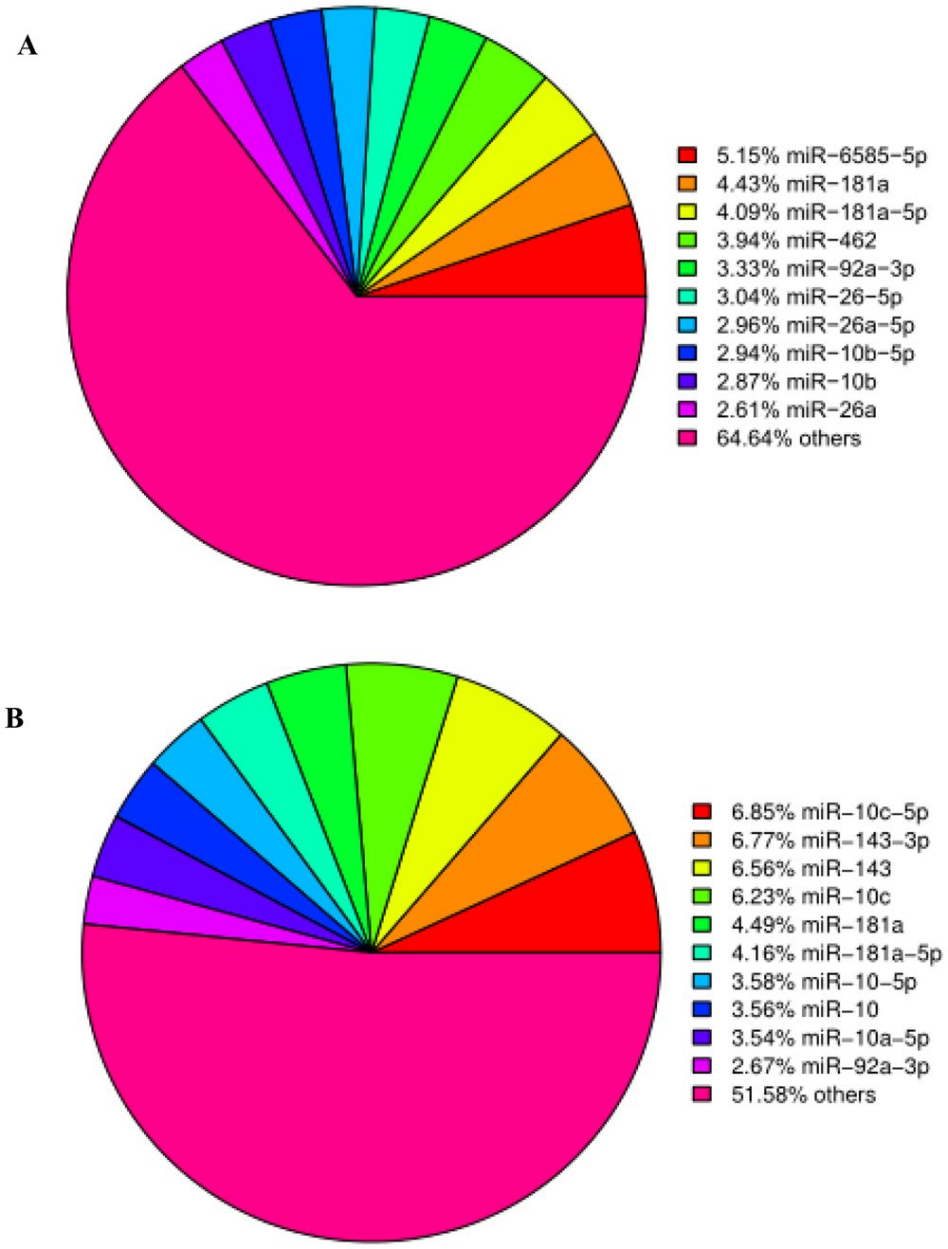
Top 10 most abundant miRNAs in the control group (CO group) (**A**) and 9.4 °C-stressed group (LTS group) (**B**) at 24 h.

The 10 most abundant miRNAs in both libraries are shown in Fig. 5. In the LTS library, miR-10c-5p, miR-143–3p, miR-143, miR-10c, miR-181a, miR-181a-5p, miR-10-5p, miR-10, miR-10a-5p, and miR-92a-3p accounted for 48.4% of the reads that mapped to sequences in miRBase. In the CO library, miR-6585-5p, miR-181a, miR-181a-5p, miR-462, miR-92a-3p, miR-26-5p, miR-26a-5p, miR-10b-5p, miR-10b, and miR-26a accounted for 35.4% of the reads that mapped to sequences in miRBase.

**Figure 6:**
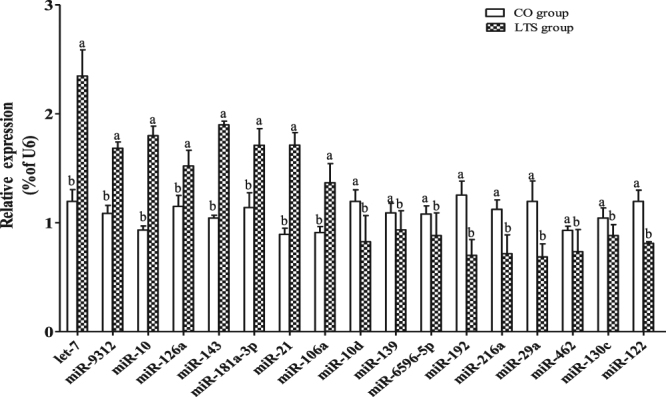
Verification of selected miRNAs between the control group (CO group) and 9.4 °C-stressed group (LTS group) at 24 h by qRT-PCR. Diverse lowercase letters show significant differences (*P* < 0.05) between the CO and LTS groups at 24 h, by Duncan’s multiple range test.

The expression profiling of the miRNAs between the CO and LTS libraries revealed 394 miRNAs that were significantly down-regulated and 189 miRNAs that were significantly up-regulated at 24 h post-9.4 °C stress. Based on transcripts with at least 100 reads in each of the libraries^35^, we identified 17 differentially expressed miRNAs, of which nine were up-regulated and eight were down-regulated (Table 3). We used qRT-PCR to validate these 17 miRNAs and found that their expression patterns were consistent with the results obtained by high-throughput sequencing (Fig. 6).

**Table 3:** Differentially expressed miRNAs in GIFT head kidney between the control group (CO group) and 9.4 °C-stressed group (LTS group). These 17 miRNAs had transcripts with at least 100 reads in each of the libraries.

miRNA	CO expression	LTS expression	Fold change (log_2_LTS/CO)	Regulation (LTS vs CO)	P-value
let-7	2299.657	6724.020	1.548	up	<0.01
miR-10	13314.634	64353.563	2.273	up	<0.01
miR-126a	1487.887	3024.875	1.024	up	<0.01
miR-143	26989.825	118048.510	2.129	up	<0.01
miR-9312	248.560	519.909	1.065	up	0.02
miR-181a-3p	1044.118	3597.701	1.785	up	<0.01
miR-21	12266.380	26068.168	1.088	up	<0.01
miR-106a	222.670	456.776	1.037	up	<0.01
miR-10d	1778.715	585.516	−1.603	down	<0.01
miR-130c	2487.338	1054.025	−1.239	down	<0.01
miR-6595-5p	696.713	199.615	−1.803	down	<0.01
miR-192	18817.615	1515.111	−3.635	down	<0.01
miR-216a	17570.678	1414.625	−3.635	down	<0.01
miR-29a	819.876	251.015	−1.708	down	<0.01
miR-462	60573.401	25337.789	−1.257	down	<0.01
miR-139	11030.362	4743.034	−1.218	down	<0.01
miR-122	2303.710	782.418	−1.558	down	<0.01

### Prediction of target genes and associated regulated pathways

**Figure 7:**
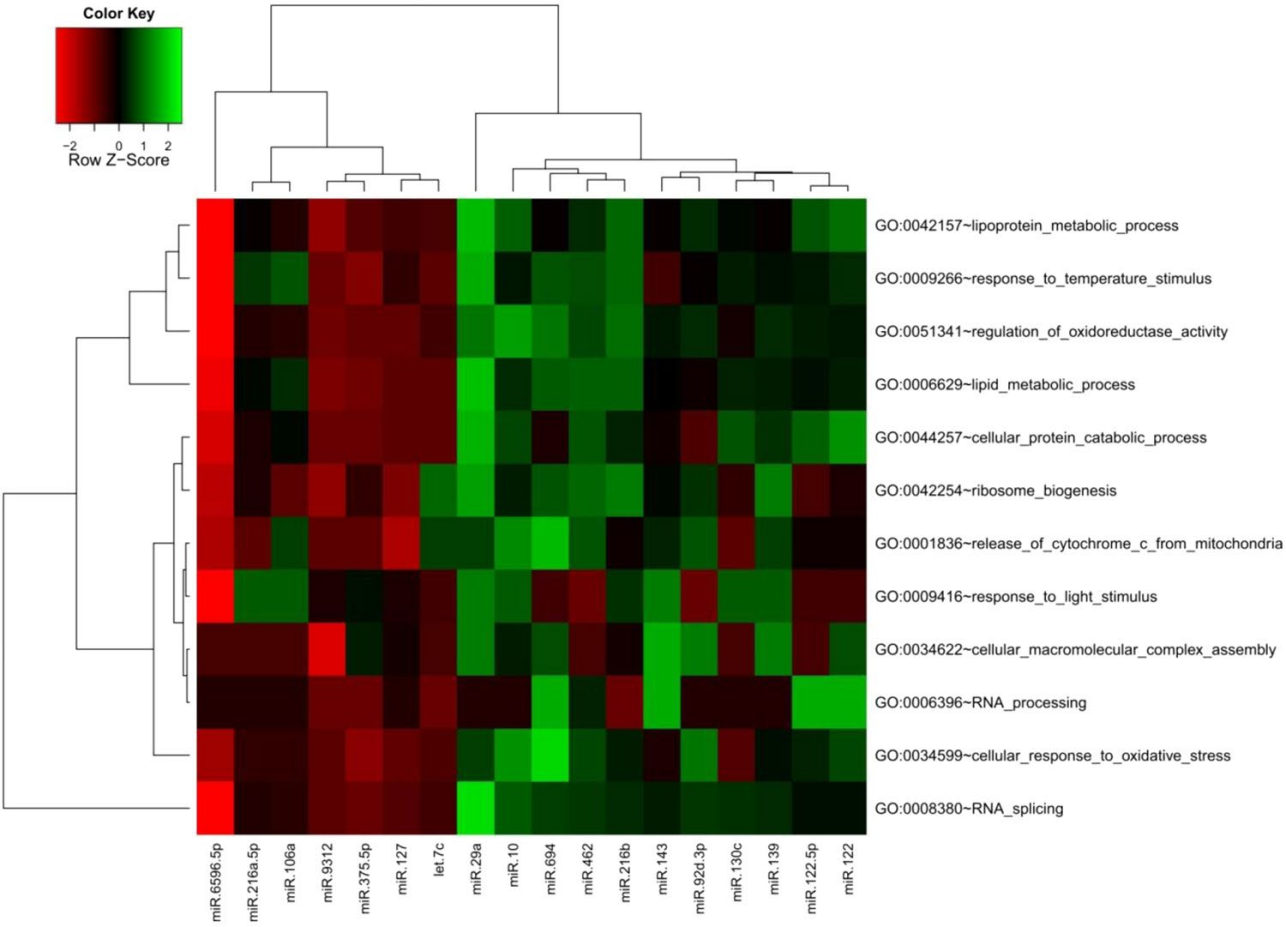
Cluster analysis of differentially expressed miRNAs in head kidney of GIFT at 24 h post-9.4 °C stress. The colour scale indicates the log2-fold change from high (red) to low (green).

To determine the possible functions of the differentially expressed miRNAs associated with cold stress, we first predicted their target genes using TargetScan 5.2 and the miRanda v3.3a toolbox. We used the gene ontology (GO) database to annotate the predicted target genes^34^. Then, we constructed a heat map based on the 12 most enriched biological pathways at 24 h under 96h-LT_50_ stress, which showed that most of the known differentially expressed miRNAs were predicted to be regulate one or more genes involved in the cold-stress response, including mainly lipid metabolic processes, response to temperature stimulus, response to oxidative stress, and cellular response to light stimulus (Fig. 7).

### Expression levels of miRNAs and their target genes in head kidney of GIFT under 96h-LT_50_ stress by qRT-PCR

**Figure 8:**
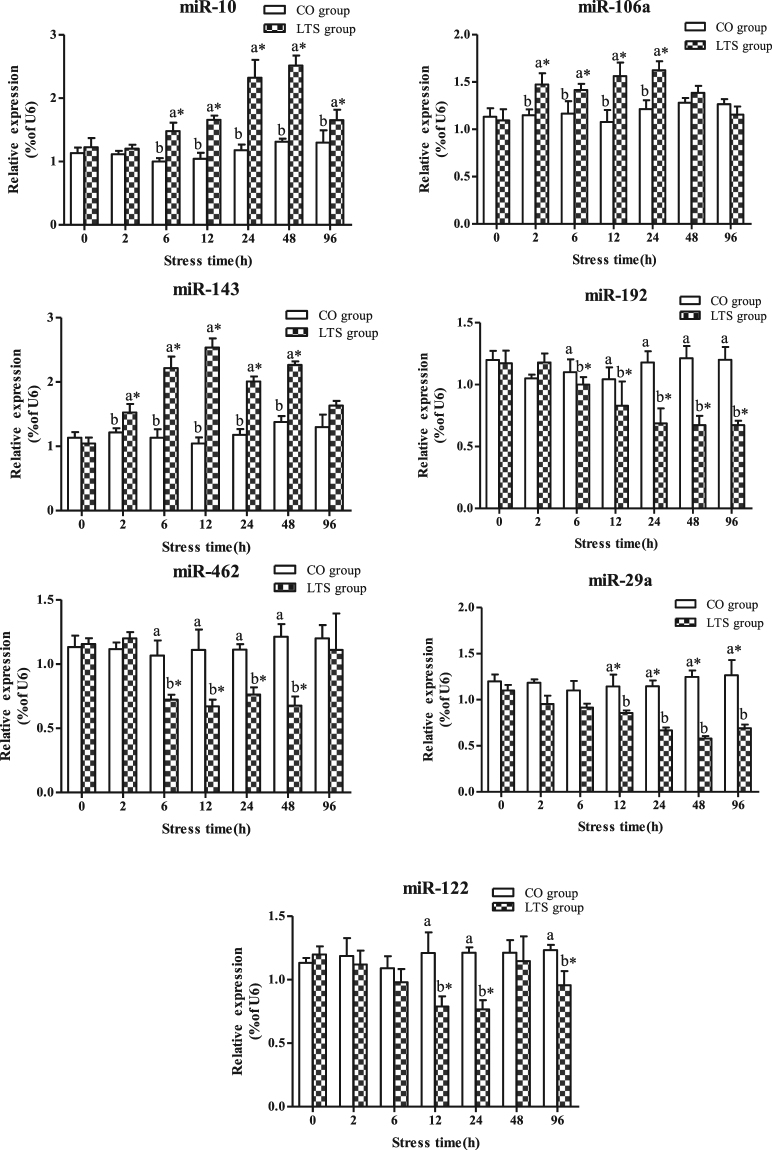
Expression levels of selected miRNAs in the control group (CO group) and 9.4 °C-stressed group (LTS group) in GIFT head kidney for 96 h. *Indicates significant differences (*P* < 0.05) between values obtained pre- and post-9.4 °C stress, by paired-samples t-test. Diverse lowercase letters show significant differences (*P* < 0.05) between the CO and LTS groups at each sampling point, by Duncan’s multiple range test.

The miR-10, miR-106a, and miR-143 expression levels first increased and then decreased under 96h-LT_50_ stress (Fig. 8). In the LTS group, the miR-10 expression level reached a peak value at 48 h and, and was significantly lower at 96 h than at 24 h and 48 h (*P* < 0.05). The miR-106a expression levels were significantly higher for the LTS group than for the CO group from 2 h to 24 h under 96h-LT_50_ stress, and the miR-143 expression levels for the LTS group were significantly higher than for the CO group from 6 h to 96 h under 96h-LT_50_ stress (*P* < 0.05). The miR-29a and miR-192 expression levels were significantly decreased from 12 h to 96 h under 96h-LT_50_ stress (*P* < 0.05). The miR-462 expression levels first decreased and then increased under 96h-LT_50_ stress, and the expression levels for the LTS group were significantly lower for the CO group from 6 h to 48 h under 96h-LT_50_ stress (*P* < 0.05). However, at 96 h under 96h-LT_50_, the expression level of miR-462 showed no significant difference between the LTS and CO groups (*P* > 0.05). Also, the miR-122 levels in the LTS group were significantly decreased at 12 h, 24 h and 96 h compared with the CO group (*P* < 0.05).

**Figure 9:**
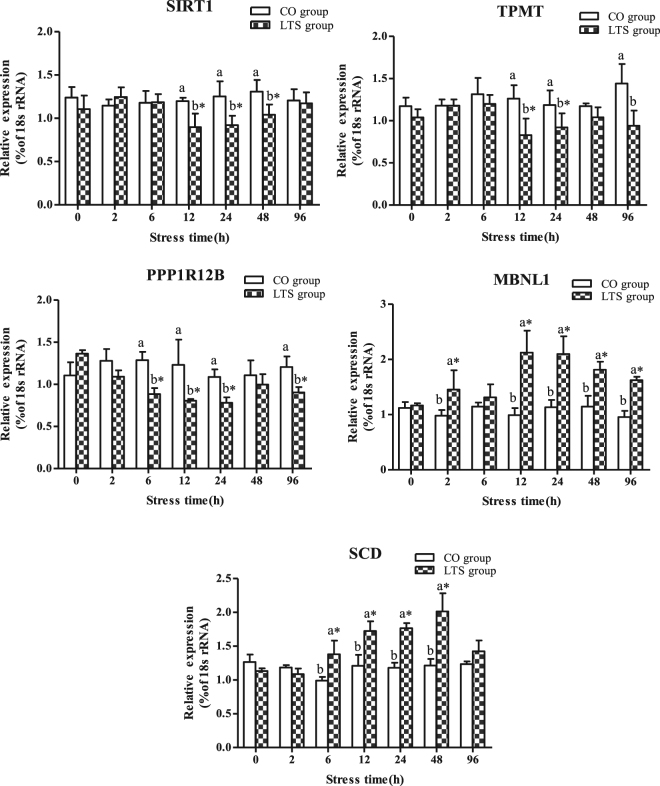
Expression levels of five potential target genes in the control group (CO group) and 9.4 °C-stressed group (LTS group) in GIFT head kidney for 96 h. *Indicates significant differences (*P* < 0.05) between values obtained pre- and post-9.4 °C stress, by paired-samples t-test. Diverse lowercase letters show significant differences (*P* < 0.05) between the CO and LTS groups at each sampling point, by Duncan’s multiple range test.

Based on the *O*. *niloticus* transcriptome sequence data, the GO and KEGG annotations, and the miRNA and mRNA expression patterns^36^, we screened and identified potential target genes of miR-10/106a/143/29a/192/462/122, which were related with cold stress. We detected negative expression levels between the following miRNA–mRNA pairs: miR-10–sirtuin 1 (*SIRT1*), miR-106a–thiopurine S-methyltransferase (*TPMT*), miR-143–protein phosphatase 1 regulatory subunit 12B (*PPP1R12B*), miR-192/462–muscleblind-like protein 1 (*MBNL1*), and miR-29a/122–stearoyl-CoA desaturase (*SCD*). The expression levels of *SIRT1*, *TPMT*, and *PPP1R12B* were significantly lower in the LTS group compared with the CO group at 12 h and 24 h, whereas the expression levels of *MBNL1* and *SCD* were significantly higher in the LTS group compared with the CO group (Fig. 9). However, at 96 h, the expression levels of *SIRT1* and *SCD* showed no significant differences between the LTS and CO groups, whereas the expression level of *MBNL1* was significantly lower than its expression level in the LTS group at 12 h and 24 h.

## Discussion

MiRNAs play important roles in regulating gene function, and the systematic identification of miRNAs and their abundance is a basis for their functional analysis. Until now, there are no reports of miRNA analysis in tilapia exposed to cold stress. Our small RNA sequencing data revealed that the 21–23 nt long reads accounted for more than 65% of the total small RNAs in the CO and LTS libraries. The proportion of common reads between the two libraries was relatively high, indicating good overall consistency in both libraries. We identified a large number of conserved miRNAs (1736 and 1481 in the CO and LTS libraries, respectively) and some novel miRNAs (164 and 152 in the CO and LTS libraries, respectively) in the head kidney of GIFT, suggesting that different species have their own sets of specific miRNAs and conserved miRNAs, and confirming that miRNAs have a faster rate of evolution than other RNA genes^37^.

Aquatic animals depend on immune regulation and cellular response to adapt to changes in environmental temperature within a certain range^5^. The miR-10 family, and miR-181a, miR-181a-5p, and let-7 are essential in mediating cell proliferation and differentiation. We found that the expression levels of members of the miR-10 family were high in the CO and LTS libraries. Several studies have shown that the miR-10 family is highly conserved among different species. Members of the miR-10 family play important regulatory roles in the development of some tumours. For example, the expression levels of miR-10b-5p and miR-10b-3p were significantly higher in the brain tissue of Huntington chorea compared with normal brain tissue^38^. The expression levels of miR-10a and miR-10b were significantly increased in invasive pituitary adenomas and were closely related to the invading degree^39^. The expression of miR-10b was positively correlated with tumour diameter of pituitary adenomas^39^. However, the role of the miR-10 family has been little reported in fish. Our high-throughput sequencing and qRT-PCR results show that, at 24 h post-9.4 °C stress, there were significant effects on the miR-10, miR-10d, and miR-10c expression levels in GIFT head kidney, indicating that these miRNAs may be involved in cellular immunity and differentiation of the head kidney post-stress. We used bioinformatics software to predict the potential target gene for miR-10, and found that the up-regulation of miR-10 may inhibit the expression of *SIRT1* mRNA. SIRT1 is a representative member of the histone deacetylase family and is involved in regulating cell metabolism, aging and apoptosis, and catalysing the deacetylation of histone, nuclear factor kappa B, and activating protein 1, thereby altering chromatin conformation, reducing transcription factor activity, and down-regulating the transcription of inflammatory genes^40^. RBA and PA have been used as the main indicators of a non-specific defence response in fish cells. In this study, the RBA and PA in head kidney macrophages of GIFT were significantly inhibited at 12 h post-9.4 °C stress. The immunosuppression induced by inhibition of *SIRT1* expression levels may have been caused by changes in cellular membrane mobility and stability of head kidney, as well as disrupting the physiological balance, which inhibited the phagocytic function of macrophages^41^.

Both miR-181a and miR-181a-5p were abundant in the two libraries. Li *et al*.^42^ showed that miR-181a played an important role in T cell differentiation and T cell receptor signalling pathways. In the serum of dairy cows under heat stress, miR-181a was significantly down-regulated^43^. The predicted target gene of miR-181a was highly enriched in the T cell and B cell receptor signalling pathway, suggesting that miR-181a may regulate tumour necrosis factor and CD38, thereby intervening in the immunity of dairy cows. Post-9.4 °C stress, the up-regulation of miR-181a-3p may be related to the cellular inflammatory response and immunoregulation of head kidney, causing the immune levels of the GIFT to decrease at 24 h post-9.4 °C stress, and leading to the observed increase in the mortality of the GIFT at 48 h and 96 h post-9.4 °C stress.

Let-7 is highly conserved in vertebrate and invertebrates, including molluscs, foci, and zebrafish^44^. Let-7 regulates brain and nervous system development in mouse^45^, and plays an important role in heart development in vertebrates. Dicer is essential for the synthesis of let-7, and Dicer knockout mice exhibit cardiac hypertrophy and dysplasia, and die from 12.5 d to 14.5 d during embryonic development^46,47^. In zebrafish embryos, Dicer knockout impaired blood circulation^46^. Johnson *et al*.^48^ found that let-7 was highly expressed in normal lung tissue, and that inhibition of let-7 increased the division of A549 lung cancer cells, whereas overexpression of let-7 in lung cancer cells affected cell-cycle progression and reduced cell division. These results suggested that the target genes of let-7 may be directly or indirectly related with cell cycle and division, thereby regulating the development of lungs. The up-regulation of let-7 in GIFT head kidney post-9.4 °C stress may alter the cellular stress response, resulting in some degree of adaptive regulation.

In mammals, miR-21, miR-139, and miR-106a are important biomarkers for cancer development^49–51^. Increased expression of miR-21 inhibited the tumour suppressor gene and increased the proliferation and invasion of prostate cancer cells^49^. Lu *et al*.^52^ and Opstad *et al*.^53^ found that miR-21 was an important regulator of delayed-type hypersensitivity and coronary artery disease, thereby regulating the interleukin-12/interferon-γ proinflammatory loop. Up-regulation of miR-139 inhibited chondrocyte proliferation and migration by regulating the mRNAs encoding eukaryotic translation initiation factor 4 gamma 2 and insulin-like growth factor 1 receptor, making miR-139 a possible therapeutic target for osteoarthritis^54^. The up-regulation of miR-21 and down-regulation of miR-139 may be associated with immunosuppressive and cell inflammatory responses in the GIFT head kidney cells after cold stress. miR-106a was found to modulate multidrug resistance in gastric, ovarian, and non-small cell lung cancers by regulating its respective target genes^51,55^. In the present study, up-regulation of miR-106a may inhibit the expression of its predicted target *TMPT*. TMPT is an intracellular enzyme that specifically catalyses the mercaptomethylation of heterocyclic and aromatic ring compounds^56^. TPMT is widely present in various tissues of the human body and has the highest activity in liver and kidney, and is also involved mainly in acute leukaemia and in the regulation of some immune diseases^56^. LYZ is an important component of the lymphocyte enzyme system in aquatic animals, and its activity reflects changes in the natural immune level of fish^57^. In this study, at 12 h post-9.4 °C stress, the down-regulation of *TMPT* mRNA levels may have an immunosuppressive effect on the adaptive adjustment of GIFT, and reduce the LYZ activity in head kidney, thereby resulting in increased mortalities.

Chu and Xu^58^ reported that increased miR-192 levels reduced the abundance of interleukin 1 receptor type I, which was involved in inflammatory and immune responses in miiuy croakers (*Miichthys miiuy*) exposed to *Vibrio anguillarum*. miR-462 played a vital role in the signal pathway of hypoxia-responsive elements in blunt snout bream (*Megalobrama amblycephala*) under hypoxia stress^59^. In this study, the down-regulation of miR-192 and miR-462 may up-regulate the expression of *MBNL1*. MBNL1 can inhibit breast cancer metastasis, which is associated with reduced clinical metastatic relfront outcomes^60^. In addition, the loss of MBNL1 function was considered to have a key role in disease, and localized adeno-associated virus delivery of *MBNL1*, as well as *MBNL1* overexpression partially rescued skeletal muscle pathology in mice with myotonic dystrophy^61^. Increased levels of *MBNL1* mRNA may help to enhance the immunization ability of GIFT head kidney and improve survival at 24 h post-9.4 °C stress.

The head kidney of GIFT may be involved in “lipid metabolic process”, and miR-130c, miR-29a, and miR-122, which were down-regulated in the LTS library, may be involved in the adaptive regulation of metabolic levels post-cold stress. When GIFT are exposed to water temperatures below the suitable temperature, internal body cold may inhibit the metabolic rate and peripheral blood flow, which could affect the integrity of tissue structure^62^. miR-130a/b were proposed as predictive biomarkers for metabolic disorders and could reflect the physiological status of cells and organs^63^. Wang *et al*.^64^ suggested that miR-130b may be involved in the pathogenesis of metabolic diseases in mouse models of obesity and in human. In addition, up-regulation of miR-130a/b was found to suppress peroxisome proliferator-activated receptor expression in the rat liver fibrosis model, and it increased the expression of extracellular matrix mRNA^64^. miR-29a and miR-122 were predicted to be involved in regulating *SCD* expression by bioinformatics analysis. In our previous study, inhibition of miR-29a stimulated *SCD* expression in GIFT fed a saturated fatty acid diet, and increased the conversion of 16:0 and 18:0 to 16:1 and 18:1^21^. After silencing of miR-122 in mouse liver, plasma triglycerides and cholesterol were significantly decreased and fat deposition was reduced^65^. Post-9.4 °C stress, down-regulation of miR-122 and miR-29a may promote the expression of *SCD* mRNA and increase the conversion from saturated fatty acids to monounsaturated and polyunsaturated fatty acids, which would help to maintain cell membrane fluidity in head kidney macrophages, and mitigate cell damage caused by the risk of rigidity^2^.

However, in our study, the expression pattern of *SCD* mRNA at 96 h post-9.4 °C stress was different from the patterns observed at 12 h and 24 h post-9.4 °C stress. This may be because many miRNAs have a common target gene, and inhibition of one miRNA may be compensated by other miRNAs, resulting in adaptive regulation^66^. In addition, the longer stress time of 96 h caused inhibition of metabolism and immune regulation, and increased cell membrane damage. The loss of cellular homeostasis is generally accompanied by the formation of ROS. Increased amounts of ROS can cause changes in the unsaturated/saturated fatty acids ratio of the phospholipid bilayer of the cell membrane and increase the production of lipid peroxides^2^. This was reflected in the increased MDA content and decreased SOD activity in GIFT post-9.4 °C stress. We found that “regulation of oxidoreductase activity” and “cellular response to oxidative stress” were associated with the important antioxidant regulatory pathway of GIFT post-9.4 °C stress. Zhao *et al*.^67^ found the miR-143 expression level was stimulated during oxidative stress in vascular smooth muscle cells, and up-regulated miR-143 was found in cardiomyocytes treated with H_2_O_2_^68^. In this study, the up-regulation of miR-143 may inhibit the expression of *PPP1R12B*. PPP1R12B has been reported to be an insulin regulating phosphatase subunit^69^. The lipids of head kidney macrophages in fish were rich in phospholipids^70^. Therefore, inhibition of *PPP1R12B* in head kidney of GIFT may be involved in the regulation of phosphorylase/phosphokinase on cell membranes. Juvenile GIFT under 96h-LT_50_ stress may not be able to produce the adaptive regulation response, which could have caused free radical metabolic disorders and lipid metabolic abnormalities, and disrupted oxidation–antioxidation homeostasis.

Two miR-126 target sites (miR-126a/b) have been detected in the zebrafish genome, and they act synergistically to regulate angiogenesis and maintain vascular integrity^71^. Head kidney tissue is an important haematopoietic organ in fish, and its integrity may help to maintain homeostasis. At 24 h post-9.4 °C stress, the inflammatory response and lipid peroxidation may damage vascular endothelial cells and vascular integrity, leading to up-regulation of miR-126a expression levels. Olena *et al*.^72^ found that inhibition of miR-216a could up-regulate sorting nexin 5 to modulate Notch signalling during zebrafish eye development. In this study, “response to light stimulus signal pathway” was enriched by cold stress. Down-regulation of miR-216a may interfere with the sensitivity of GIFT to light or cause eye damage and reduce balance capacity, which may explain the accumulation of GIFT at the bottom of the tank after exposure to 96h-LT_50_ stress. We could not find any reports about the possible roles of miR-9312 and miR-6596-5p. The heat map analysis showed that these two miRNAs may be associated mainly with “response to light stimulus” and “RNA processing” pathways. In future experiments, we plan to study the signal pathways that involve the target genes of these two miRNAs.

In conclusion, we first analysed the physiological responses and miRNA profiles of GIFT head kidney under 96h-LT_50_ cold stress. Seventeen differentially expressed miRNAs were found between the CO and LTS libraries, including nine up-regulated and eight down-regulated miRNAs. Five potential target genes were identified and their functional characteristics showed they were involved mainly in signalling pathways, including “lipid metabolic processes”, “response to immune regulation and cellular response”, and “response to oxidative stress”. The 96h-LT_50_ cold stress may have led to free radical metabolic disorders as a result of excessive oxidative stress, increased MDA content, and lipid metabolism dysfunction, resulting in imbalance of the antioxidant system. In addition, the immune responses of GIFT head kidney cells were inhibited, and the LYZ activity, and RBA and PA were significantly decreased under 96h-LT_50_ cold stress, eventually leading to increased mortality. In future studies, we will further investigate the relationship among the GIFT miRNAs and their target genes, and use a proteome profiler to better understand the molecular regulatory networks associated with the cold-stress response. Furthermore, the tolerance of fish to cold stress may be different at different developmental stages. Therefore, we will study the biological response characteristics of cold-stressed fish at different developmental stages to obtain more information about the adaptation and regulation mechanisms in teleost.
